# Cardiometabolic risk factors and health behaviors in family caregivers

**DOI:** 10.1371/journal.pone.0176408

**Published:** 2017-05-04

**Authors:** Alyson Ross, Robert Shamburek, Leslie Wehrlen, Stephen D. Klagholz, Li Yang, Elyssa Stoops, Sharon L. Flynn, Alan T. Remaley, Karel Pacak, Nonniekaye Shelburne, Margaret F. Bevans

**Affiliations:** 1 Nursing Department, Clinical Center, National Institutes of Health, Bethesda, Maryland, United States of America; 2 National Heart, Lung, and Blood Institute, National Institutes of Health, Bethesda, Maryland, United States of America; 3 Department of Laboratory Medicine, Clinical Center, National Institutes of Health, Bethesda, Maryland, United States of America; 4 National Institute of Child Health and Human Development, National Institutes of Health, Bethesda, Maryland, United States of America; 5 National Cancer Institute, National Institutes of Health, Bethesda, Maryland, United States of America; University of Insubria, ITALY

## Abstract

The purpose of this study was to compare components of cardiometabolic risk and health behaviors of 20 family caregivers of allogeneic hematopoietic stem cell transplant patients to those of age, gender, and race/ethnicity-matched controls. A prospective, repeated measures design was used to compare cardiometabolic risk and health behaviors in caregivers and controls at three time-points: pre-transplantation, discharge, and six weeks post-discharge. Measures included components of metabolic syndrome, Reynolds Risk Score, NMR serum lipoprotein particle analyses, and the Health-Promoting Lifestyle Profile II (HPLP-II). Mixed-model repeated measure analyses were used. There were no between or within group differences in LDL cholesterol, HDL cholesterol, and triglycerides. There was a significant interaction effect between time and role in large VLDL concentration (VLDL-P) (F (2, 76) = 4.36, *p* = .016), with the trajectory of large VLDL-P increasing over time in caregivers while remaining stable in controls. Within caregivers, VLDL particle size (VLDL-Z) was significantly larger at time-point three compared to time-points one (*p* = .015) and two (*p* = .048), and VLDL-Z was significantly larger in caregivers than in controls at time point three (*p* = .012). HPLP-II scores were lower in caregivers than controls at all time-points (*p* < .01). These findings suggest that caregiving may have a bigger impact on triglycerides than on other lipids, and it is through this pathway that caregivers may be at increased cardiometabolic risk. More sensitive measurement methods, such as NMR lipoprotein particle analyses, may be able to detect early changes in cardiometabolic risk.

## Introduction

Exposure to prolonged stress is associated with increased morbidity and mortality, primarily from cardiovascular disease (CVD) [1]. Although the exact pathway whereby chronic stress leads to CVD is unclear, it appears that heightened activation or dysregulation of the hypothalamic-pituitary-adrenal axis and the sympathetic nervous system [2,3] may be contributing factors that result in endothelial dysfunction [4]. Individuals who engage in healthy activities such as physical activity, rest and relaxation, social connection, proper nutrition, as well as refraining from tobacco and excessive alcohol consumption, may be protected from some of the damaging effects of chronic stress [2]. Unfortunately, unmanaged stress negatively impacts one’s health behaviors, as individuals who are stressed are less likely to engage in regular exercise, and more likely to smoke, ingest excessive amounts of alcohol, and consume foods high in sugar, fat, and salt, all of which contribute to cardiometabolic diseases such as CVD and type 2 diabetes [5].

The stress and strain of being a caregiver increases one’s all-cause risk of mortality [6], although there is some controversy whether this increased risk is a direct result of caregiving or whether the general stress of having a severely ill loved one is the cause of this increased morbidity of mortality [7]. However when evaluating the evidence specific to the effect of caregiving on cardiovascular health, the findings are more consistent. Findings from the Nurses’ Health Study (NHS) examining 54,412 women with no baseline evidence of CVD suggested that women who cared for a disabled or ill spouse for 9+ hours per week had an age-adjusted relative risk of CVD of 1.93 (95% CI, 1.16–3.20) compared with non-caregivers [8]. Additionally, individuals providing care to a spouse with Alzheimer’s disease exhibited increased Framingham Coronary Heart Disease scores compared to controls (8.0 ± 2.9 vs. 6.3 ± 3.0 (*p* = .013)), with hypertension making the largest contribution to their CVD risk [9]. Much of the prior research on CVD risk in caregivers has been conducted in dementia caregivers and has focused on hypercoagulability as a potential mechanism; dementia caregivers have demonstrated greater increases in the pro-coagulant factor D-dimer and tissue plasminogen activator (t-PA) antigen [10]. In addition, dementia caregivers had a 2.2 times greater odds of having carotid artery plaque measured via B-mode ultrasonography compared with non-caregivers, even when controlling for other CVD risk factors (95% CI, 1.10–4.73, *p* = .48) [11].

While caregiving appears to increase the risk of morbidity and mortality, particularly from CVD, nearly all of the research has focused on caregivers of individuals with dementia. No published studies were found examining cardiometabolic risk factors in caregivers of patients undergoing allogeneic hematopoietic stem cell transplantation (HSCT). In the realm of cancer care, HSCT is one the most intense cancer treatments, potentially leading to serious and sometimes life-threatening treatment toxicities, as well as long-term health issues from chronic graft versus host disease [12]. Accordingly, transplant centers require transplant patients to have a caregiver at all times during the immediate post-transplant recovery period to provide physical and emotional support to the patient and to assist in the assessment and management of treatment-related symptoms [13]. While caregiving can be a rich and rewarding experience [14], individuals providing care to a friend or family member undergoing HSCT experience a multitude of competing demands that can lead to disruption in their daily lives and profound stress [15–17]. Caregivers of HSCT patients typically experience high levels of caregiver burden and, compared to age, gender, and race/ethnicity matched controls, they experience prolonged and significantly higher levels of stress, anxiety, and depression [18,19].

While the impact of caregiving on psychosocial aspects of caregivers’ health is well described, the effects on health behaviors are less well understood. Evidence shows that HSCT caregivers will prioritize the needs of the patient over their own [20]. Cancer caregivers report modifying their lifestyle to accommodate their loved one’s needs, including restricting physical activity and leisure time, as well as losing contact with their social support network of family and friends [21]. However, the evidence is inconclusive regarding whether cancer caregiving is associated with decreases in healthy behaviors that are cardio-protective, such as physical activity, proper nutrition, social interaction, and stress reduction [22].

Determining cardiometabolic risk is complex and includes a variety of individual factors that are often used in clinical practice within composite measures. Two common composite measures are metabolic syndrome and the Reynolds Risk Score. Metabolic syndrome, a cluster of symptoms that place individuals at increased risk for CVD [23], has been defined by the Third Report of the National Cholesterol Education Program Adult Treatment Panel’s as the presence of three or more of the following: hyperglycemia (fasting blood glucose ≥ 110 mg/dL), hypertension (blood pressure (BP) ≥ 130/85 mmHg), hypertriglyceridemia (serum triglycerides > 150 mg/dL), low high-density lipoprotein cholesterol (HDL-C) < 40 mg/dL in men and < 50 mg/dL in women, and waist circumference > 40 inches in men and > 35 inches in women [24]. The World Health Organization (WHO) also includes inflammation, measured as hs-CRP, as a component of metabolic syndrome, and the addition of hs-CRP enhances the predictive values for cardiovascular risk [25]. The Reynolds Risk Score utilizes traditional measures from the Framingham Risk Score (FRS) including: age, gender, total cholesterol, HDL-C, systolic blood pressure (SBP), smoking status, and antihypertensive medication usage to calculate an individual’s 10-year risk of CVD [26]. However, the Reynolds Risk Score also includes family history and hs-CRP, potentially providing better prediction of 10-year CVD risk than the FRS [27].

Low-density lipoprotein cholesterol (LDL-C) has also been recognized as an important predictor of cardiometabolic risk, and reducing LDL-C with statins improves morbidity and mortality [28]. Recently, nuclear magnetic resonance (NMR) spectroscopy, has become an established technique for quantifying both the concentration and size of serum lipoproteins, and measurements of total LDL particle concentration (LDL-P) and particle size (LDL-Z) may serve as more accurate, sensitive indicators of true cardiometabolic risk than measurements of LDL-C [29].

The purpose of these analyses is to compare over time the cardiometabolic risk and health behaviors of HSCT caregivers to age, gender and race/ethnicity matched controls. The primary research question was: Are there differences in HSCT caregivers compared with controls in measures of cardiometabolic risk (components of metabolic syndrome, Reynolds Risk Score, and NMR serum lipoprotein particle concentration and size), as well as differences in health behaviors that contribute to cardiometabolic risk? A secondary research question was: Do health behaviors predict cardiometabolic risk in HSCT caregivers?

## Methods

### Study design and participants

This study utilized a prospective repeated measure design to compare changes in components of cardiometabolic risk and health behaviors in 20 family caregivers of adults undergoing HSCT. Data were collected at three time-points: pre-transplantation (pre-HSCT), time of initial hospital discharge (±1 week), and six weeks post-discharge (6wk) (±1 week) compared with age-, gender-, and race/ethnicity matched controls. This study was approved by the National Heart, Lung, and Blood Institute intramural Institutional Review Board and written informed consent was obtained for all participants. The study design, participants and procedures have been described in detail previously in a manuscript that examined biomarkers of stress [19]. Caregivers of adult patients receiving their first HSCT at the National Institutes of Health (NIH) Clinical Center were approached prior to the patient’s transplant and were enrolled if they were able to read and write in English or Spanish and ≥ 18 years of age. The NIH Clinical Center Clinical Research Volunteer Program registry was used to identify 20 gender, age (± 10 years) and race/ethnicity-matched controls meeting the same inclusion criteria. Exclusion criteria that were the same for both groups included: glucocorticoid treatment in the last two months; diagnosis of Cushing, Addison or Parkinson’s disease; heart transplant; pacemaker; orthostatic hypotension or autonomic dysfunction; unwilling to cease smoking for at least 12 hours or drinking alcohol for at least 24 hours prior to specimen collection; or serving as a paid caregiver for an individual. Control subjects were also excluded if they were currently receiving psychiatric care/medications, had served as a family caregiver in the preceding three months, or had experienced a substantially stressful life event in the preceding three months.

### Measures

At each time-point, caregiver and control subjects were asked to complete a demographic and health behavior questionnaire, as well as a clinical review, including a physical exam, health history, and twelve-hour fasting blood samples. These visits incorporated a determination of Eastern Cooperative Oncology Group (ECOG) performance status (Eastern Cooperative Oncology Group, 2002) and completion of the Charlson Comorbidity Index [30], a review of recent stressful life events, and anthropomorphic measurements (height, weight, BP, heart rate, waist circumference, and BMI). Height and weight measurements were obtained with subjects wearing light clothing, without shoes, and rounded to the nearest 0.1 centimeter and kilogram, respectively. Waist circumference was measured at the level of the umbilicus at the end of exhalation, with the subject lying down. BP was measured by a Dinamap^®^ Pro 100-400v2 electronic BP machine on either the right or left upper arm of the subject, following a 5-minute rest period in a sitting position; three BP measurements were obtained and the average was used. Blood samples were obtained from an indwelling catheter placed in the subject’s arm and run in a clinical chemistry panel (total cholesterol, HDL-C, LDL-C, triglycerides, glucose, and hs-CRP), as well as analyzed for NMR serum lipoprotein particle concentration and size. Clinical chemistry panels were obtained through an enzymatic immunoassay using a Roche Cobas^®^ 6000 Analyzer (Roche Diagnostics, Indianapolis, IN).

#### Cardiometabolic risk

Cardiometabolic risk is measured in this study using: components of metabolic syndrome, Reynolds Risk Score, LDL-C, and NMR serum lipoprotein particle concentration and size.

The following components of metabolic syndrome were used: SBP, DBP, waist circumference, and fasting levels of serum glucose, triglycerides, HDL-C, and hs-CRP. While related to cardiometabolic risk but not specifically listed as components of metabolic syndrome, an apolipoprotein panel and LDL-C were also obtained, and a Homeostasis Model Assessment of Insulin Resistance (HOMA-IR) was calculated.

A Reynolds Risk Score was calculated for each subject [26]. The score was calculated using each subject’s: age, current smoking status, BP, total cholesterol, HDL-C, hs-CRP, and familial history of myocardial infarction. The score is calculated and stratified into one of four risk categories: Low risk (< 5% chance), low to moderate risk (5% to 10% chance), moderate to high risk (10% to 20% chance), or high risk (20% or higher chance).

NMR spectroscopy was used as a technique to quantify both the concentration and size (diameter) of serum lipoproteins. Aliquots of frozen serum samples were used for the NMR analysis. LDL-P, LDL-Z, high-density lipoprotein particle concentration (HDL-P) and size (HDL-Z), and very low-density lipoprotein particle size (VLDL-Z) were calculated from the amplitudes of their spectroscopically unique lipid methyl group NMR signals. Relative mass percentage was also quantified from methyl group signals, which gave estimations of concentrations and sizes of lipoprotein subclass by size (small LDL-P, large HDL-P, and large VLDL-P). Prior studies have shown that measurements of total LDL-P and LDL-Z may serve as more accurate indicators of true cardiovascular risk than LDL-C when LDL-C and LDL-P are discordant, especially because cholesterol content of LDL varies greatly among individuals [29,31,32]. Lipid subclass profiles were obtained using the Vantera^®^ Clinical Analyzer (LipoScience, Inc., Raleigh, NC) at the NIH Clinical Center’s Department of Laboratory Medicine.

#### Health behaviors

Each subject was screened for their current and past alcohol and tobacco usage at each clinic visit. Tobacco frequency and duration of consumption was assessed and documented based on the number of tobacco products consumed per day and their lifetime pack year history.

The Health-Promoting Lifestyle Profile II (HPLP-II) is a self-administered 52-item instrument that measures the frequency of self-reported healthy behaviors using a 4-point Likert scale [33]. It consists of 6 subscales: Physical activity, spiritual growth, health responsibility, interpersonal relations, nutrition, and stress management. Scores range from 52 to 208, with higher scores indicating more frequent engagement in health behaviors. Cronbach’s alphas for the total scale were .97 at all three time-points and subscale coefficients ranged from .80 to .95.

### Statistical analysis

The distributions of all variables at each time-point were examined and appropriate descriptive statistics were computed (e.g., mean and standard deviation (SD) for interval level data, median for ordinal level data, frequency and percentage for categorical data). Variables were natural log or square root transformed in the final model if necessary to meet normality assumption for the analyses. Baseline between group differences were evaluated using appropriate parametric or non-parametric tests (Chi-square test, Fisher’s exact test, t test, or Wilcoxon signed rank test).

Mixed model repeated measure analyses were used to determine whether there was a change in all cardiometabolic factors and health behaviors over three study time-points between groups. Visit was treated as a categorical variable. Restricted maximum likelihood (REML) procedure was used for model parameter estimation. Aikake information criterion (AIC) and Bayesian information criterion (BIC) were used to compare and select models. Demographic variables including income and education were tested and added as covariates in the final model if they were significant predictors or contributed to the model. All data analyses were performed using SAS version 9.3 (SAS Institute Inc., Cary, NC). A *p* < .05 was considered significant.

## Results

Baseline demographic characteristics, cardiometabolic risk factors, and health behaviors for both caregivers and control subjects are detailed in Table 1. A more detailed description of the control subjects, caregiver subjects and their transplant recipients has been published previously [19]. The patients were on average 20 months (±21.2 months; range 2–76) from initial diagnosis. Twenty-eight of 40 eligible caregivers enrolled, and 21 completed the study [19]. Control subjects reported higher levels of income (*p* = .02) and education (*p* = .01) than caregivers at baseline. Otherwise, there were no significant differences in the two groups in demographic variables.

**Table 1:** Demographics, cardiometabolic risk factors and health behaviors at baseline.

	Caregivers n = 21	Normal Controls n = 20
Demographics	M (SD) min-max	
Age, years	52.2 (11.4) 30.5–74	51.1 (11.0) 29.9–74
Gender, female, n (%)	12 (57.1)	11 (55.0)
Education, n (%)^a^^b^**		
High school or less	3 (15.0)	0 (0)
Associate’s degree/some college	9 (45.0)	2 (10.5)
Bachelor’s degree	3 (15.0)	10 (52.6)
Graduate or professional degree	5 (25.0)	7 (36.8)
Income, n (%)**		
<$10,000	6 (28.6)	1 (5.0)
$10,000-$29,999	1 (4.8)	1 (5.0)
$50,000-$69,999	4 (19.0)	1 (5.0)
$70,000-$89,999	3 (14.3)	4 (20.0)
$90,000-$149,999	4 (19.0)	7 (35.0)
More than $150,000	3 (14.3)	6 (30.0)
**Cardiometaolic Risk Factors**		
BMI, kg/m^2^	28.7 (4.4) 20.8–36.8	27.3 (5.3) 20–36.1
SBP, mmHg	115.4 (10.6) 98–143	115.3 (10.2) 96–137
DBP, mmHg	68.6 (6.6) 56–85	68.8 (6.6) 58–80
Hypertension^c^, n (%)	6 (28.6)	3 (15)
Total Cholesterol, mg/dL	170.2 (28.9) 97–208	172.7 (25.0) 135–232
HDL-C, mg/dL	50.1 (16.4) 21–77	52.6 (10.9) 27–69
LDL-C, mg/dL	94.0 (22.9) 45–133	101.9 (20.6) 69–134
Triglycerides, mg/dL	130.0 (90.0) 43–349	94.8 (55.3) 39–244
Hypertriglyceridemia^d^, n (%)	7 (33)	2 (10)
Glucose, mg/dL	92.6 (7.5) 83–106	94.7 (9.9) 80–123
Hyperglycemia^e^, n (%)	4 (19)	1 (5)
Apolipoprotein A1, mg/dL	145.3 (28.7) 98–209	146.7 (19.9) 118–207
Apolipoprotein B, mg/dL	84.9 (20.0) 44–122	85.1 (12.7) 69–111
Apolipoprotein B/A1 ratio	0.6 (0.2) 0.3–1.0	0.6 ((0.1) 0.5–0.8
HOMA-IR	3.6 (6.3) 0.8–29.5	2.4 (2.9) 0.5–12.9
hs-CRP, mg/L	2.1 (2.0) 0.2–6.2	1.4 (1.4) 0.2–5
Metabolic Syndrome, yes, n (%)	6 (28.6)	2 (10.0)
Reynolds Risk Score, n (%)		
Low risk, <5%	16 (76.2)	18 (90.0)
Low to Moderate risk, 5%-10%	3 (14.3)	2 (10.0)
Moderate to High risk, 10%-20%	2 (9.5)	0
High risk, >20%	0	0
LDL-P, nmol/L	905.9 (173.3) 534–1250	925.1 (178.7) 660–1253
HDL-P, μmol/L	19.9 (3.0) 15.2–28.6	20.1 (2.4) 13.7–24.1
Small LDL-P, nmol/L^a^^b^	276.2 (212.19) 70–657	185.25 (118.9) 27–423
Large HDL-P, μmol/L	2.3 (1.3) 0.8–5	2.3 (1.2) 0.7–4.8
Large VLDL-P nmol/L	3.9 (3.0) 0.5–10.7	3.04 (2.3) 0.3–8.1
LDL-Z, nm*	21.1 (1.0) 19.7–22.5	21.7 (0.8) 20.1–22.9
HDL-Z, nm	9.5 (0.7) 8.4–11.1	9.6 (0.8) 8.2–11
VLDL-Z, nm	52.1 (5.1) 43.3–63.4	50.4 (5.9) 37.8–62.5
**Health Behaviors**		
Alcohol use, yes, n (%)	13 (61.9)	18 (90.0)
Tobacco use, yes, n (%)	3 (14.3)	2 (10.0)
Health-Promoting Lifestyle Total***	2.4 (0.6) 1.1–3.8	2.9 (0.4) 1.9–3.7
Nutrition	2.6 (0.7) 1.1–3.8	2.8 (0.6) 1.4–3.7
Physical Activity***	1.9 (0.8) 1.0–4.0	2.6 (0.9) 1.3–3.9
Interpersonal Relations***	2.7 (0.7) 1.1–3.8	3.2 (0.5) 2.1–3.9
Spiritual Growth***	2.8 (0.7) 1.2–3.8	3.4 (0.4) 2.6–4
Stress Management***	2.2 (0.7) 1–4	2.8 (0.5) 1.6–3.6
Health Responsibility***	2.1(0.6) 1.1–3.7	2.7 (0.6) 1.4–4

*M* mean, *SD* standard deviation, *BMI* body mass index, *SBP* systolic blood pressure, *DBP* diastolic blood pressure, *HDL-C* high-density lipoprotein cholesterol, *LDL-C* low-density lipoprotein cholesterol, *hs-CRP* high-sensitivity C-reactive protein, *HOMA-IR* homeostatic model assessment for insulin resistance, *Reynolds Risk Score* Percent risk of a cardiac event in the next 10 years, *LDL-P* low-density lipoprotein particle number, *HDL-P* high-density lipoprotein particle number, *VLDL-P* very low-density lipoprotein particle number, *LDL-Z* low-density lipoprotein particle diameter, *HDL-Z* high-density lipoprotein particle diameter, *VLDL-Z* very low-density lipoprotein particle diameter.

^a^n = 20 caregivers,

^b^n = 19 normal controls,

^c^BP > 130/85 or antihypertensive medication,

^d^Triglycerides > 150 mg/dl or lipid-lowering medication,

^e^Glucose >110 mg/dl or insulin or oral hypoglycemic medication

*p < .05,

**p < .02,

***p < .01

At baseline, the majority of subjects in both groups were overweight (n = 8, 38.1% caregivers; n = 3, 15% controls) or obese (n = 7, 33.3% caregivers; n = 8, 40% controls) and had at least one chronic health condition (n = 16, 76.2% caregivers; n = 15, 75% controls). There were no significant group differences in the use of medications for diabetes (*p* = 0.169) or CVD (hypertension and/or hypercholesterolemia) (*p* = 0.159). Approximately one quarter of the caregivers (n = 6, 28.6%) and 10% (n = 2) of control subjects met the criteria for metabolic syndrome, but the large majority of both the caregivers (n = 16, 76%) and the controls (n = 17, 89%) had Reynold’s Risk Scores that placed them at low risk for CVD. The NMR analysis revealed mean values of HDL-P and large HDL-P placed both caregivers and controls in the higher cardiometabolic risk category, while levels of small LDL-P, large VLDL-P, and VLDL-Z placed both groups at moderate risk [32,34,35]. The sole baseline difference between the two groups in cardiometabolic risk was LDL-Z, which was significantly smaller in the caregivers than the controls (*p* = .036).

**Fig 1:**
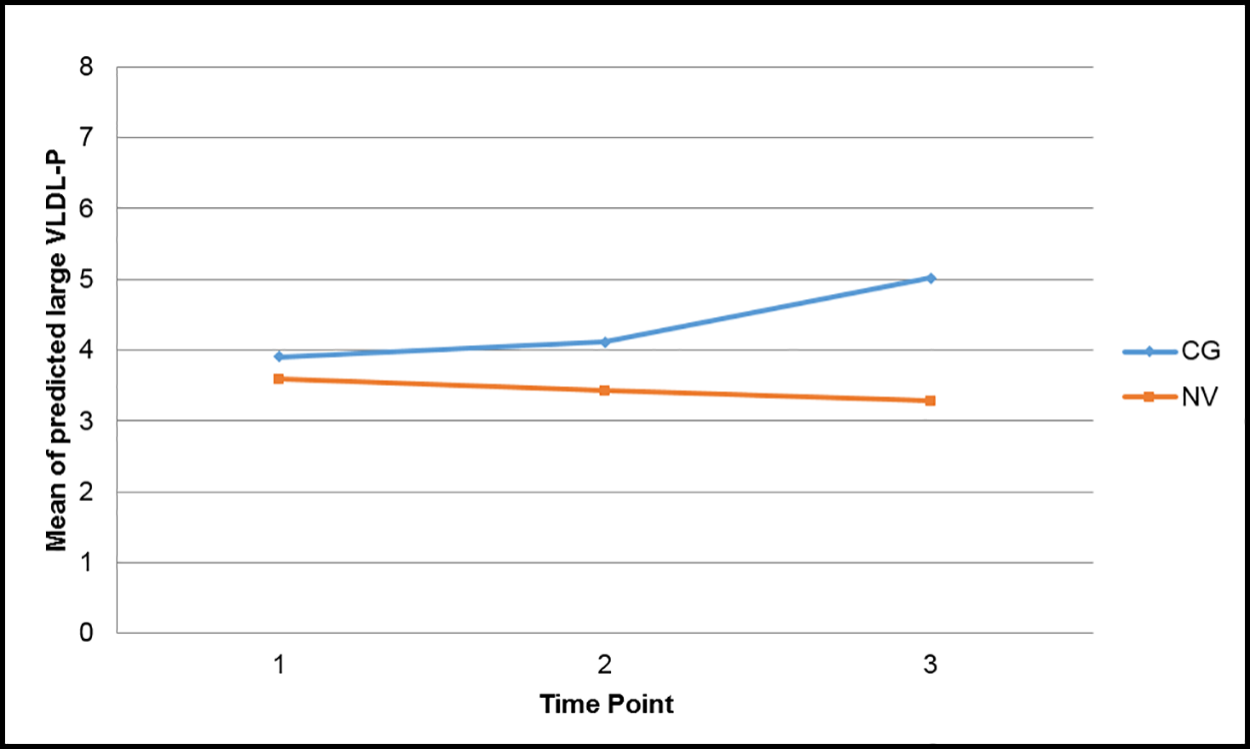
Predicted large VLDL-P particle concentration over time based on caregiver status, controlling for income. Contrast tests showed significant differences between time-point 3 and time-point 1 (*p* = .002) and between time-point 3 and time-point 2 (*p* = .0112) for caregiver group only. *CG* caregiver, *NV* normal volunteer.

**Fig 2:**
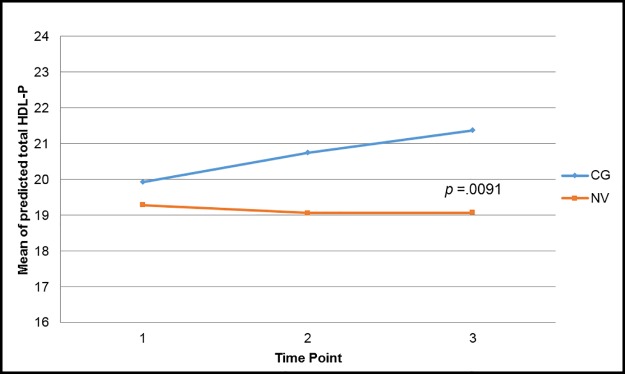
Predicted total HDL-P particle concentration over time based on caregiver status, controlling for income. Contrast tests showed significant differences between time-point 3 and time-point 1 for caregiver group only (*p* = .0017). *CG* caregiver, *NV* normal volunteer.

**Fig 3:**
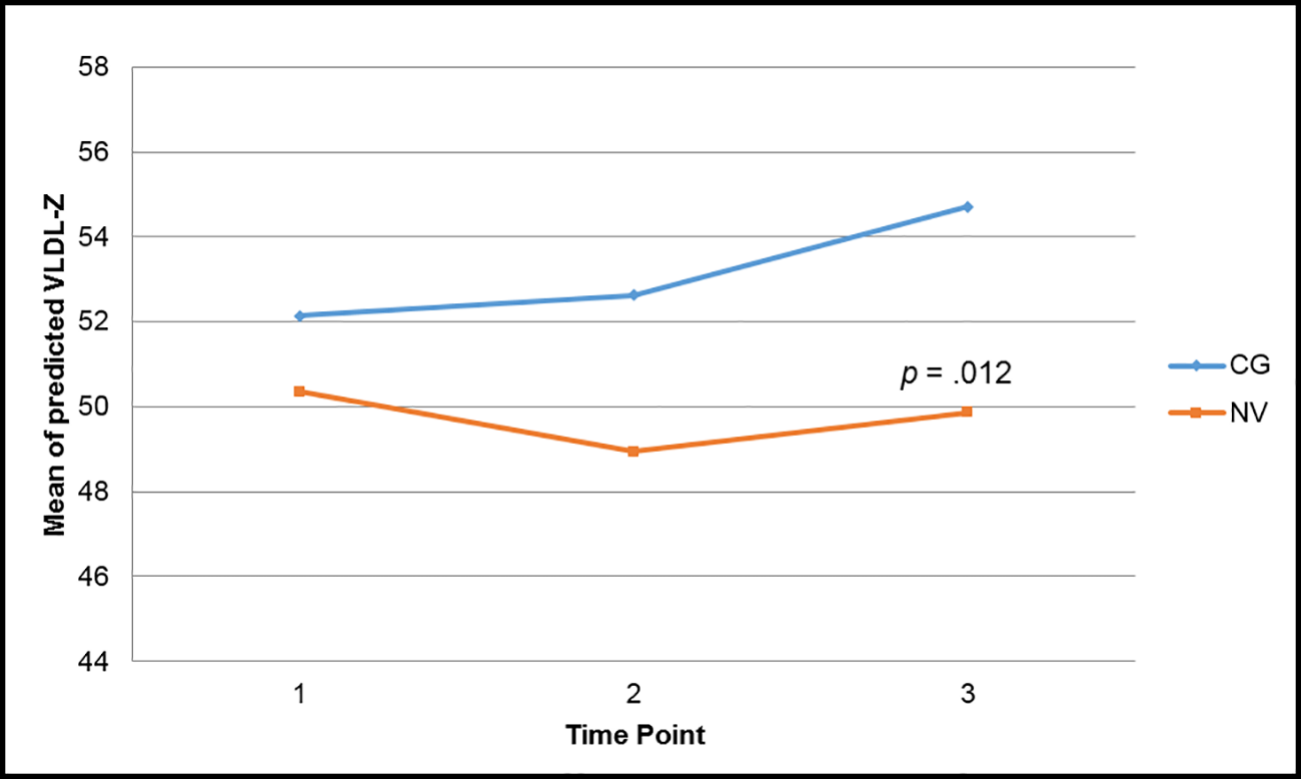
Predicted total VLDL-Z particle size over time based on caregiver status. Contrast tests showed significant differences between time-point 3 and time-point 1 (*p* = .0152) and between time-point 3 and time-point 2 (*p* = .0481) for caregiver group only. *CG* caregiver, *NV* normal volunteer.

Examining between and within group effects, there were no significant effects in any of the traditional markers of cardiometabolic risk in the final models. However, there were significant effects in three of the lipoprotein particle size and concentration markers of cardiometabolic risk. Estimated mean differences between the two groups for the main outcomes are shown in Table 2. First, controlling for income, there was a significant interaction effect between time and role in large VLDL-P (F (2, 76) = 4.36, *p* = .016), with trajectories of large VLDL-P increasing over time in caregivers compared with controls (Fig 1). Caregiver within-group analyses revealed that levels of large VLDL-P were significantly higher at time-point three compared with time-point one (*p* = .002) and time-point two (*p* = .011). Second, when controlling for income, there was a significant interaction effect between time and role in HDL-P (F (2, 76) = 3.49, *p* = .035), with levels of HDL-P increasing in caregivers while HDL-P in controls were stable; time-point three was significantly higher than time-point one in the caregivers (*p* = .002), and this level was significantly higher than controls (*p* = .009) (Fig 2). Finally, there was a significant main effect of role in levels of VLDL-Z (F (1, 39) = 4.28, *p* = .045), with levels of VLDL-Z significantly higher in caregivers than in control subjects at time-point three (*p* = .012) and within the caregivers, levels of VLDL-Z at time-point three were significantly higher than at time-points one (*p* = .015) and two (*p* = .048) (Fig 3).

**Table 2:** Estimated mean differences between caregivers and controls for the main outcomes, according to mixed model repeated measures analysis.

Outcome	Baseline	Discharge	6-Weeks Post
		Mean difference (95% CI)	
HDL particle^a^	0.65 (-1.05, 2.35)	1.69 (-0.01, 3.39)	2.31 (0.59, 4.03)**
Large VLDL-P^a^	0.31 (-1.61, 2.24)	0.68 (-1.24, 2.61)	1.74 (-0.20, 3.67)
VLDL diameter	1.79 (-1.92, 5.49)	3.69 (-0.01, 7.39)	4.84 (1.09, 8.58)*
HPLP total	-0.53 (-0.85, -0.21)**	-0.50 (-0.80, -0.19)**	-0.54 (-0.82, -0.27)***
Physical activity	-0.74 (-1.26, -0.21)**	-0.58 (-1.05, -0.11)*	-0.59 (-1.04, -0.13)*
Relationships	-0.53 (-0.91, -0.16)**	-0.58 (-0.94, -0.21)**	-0.53 (-0.91, -0.15)**
Spirituality	-0.55 (-0.90, -0.19)**	-0.50 (-0.85, -0.14)**	-0.51 (-0.89, -0.12)*
Stress reduction	-0.57 (-0.95, -0.19)**	-0.60 (-0.96, -0.25)**	-0.71 (-1.04, -0.37)***
Health responsibility	-0.60 (-1.00, -0.21)**	-0.50 (-0.89, -0.12)*	-0.58 (-0.95, -0.21)**

All differences were calculated as caregiver values minus control values.

*CI* Confidence Interval, *HPLP* Health-Promoting Lifestyle Profile II (HPLP-II).

^a^Income was controlled in the model.

*p < .05,

** *p <* .01,

*** *p <* .001

**Fig 4:**
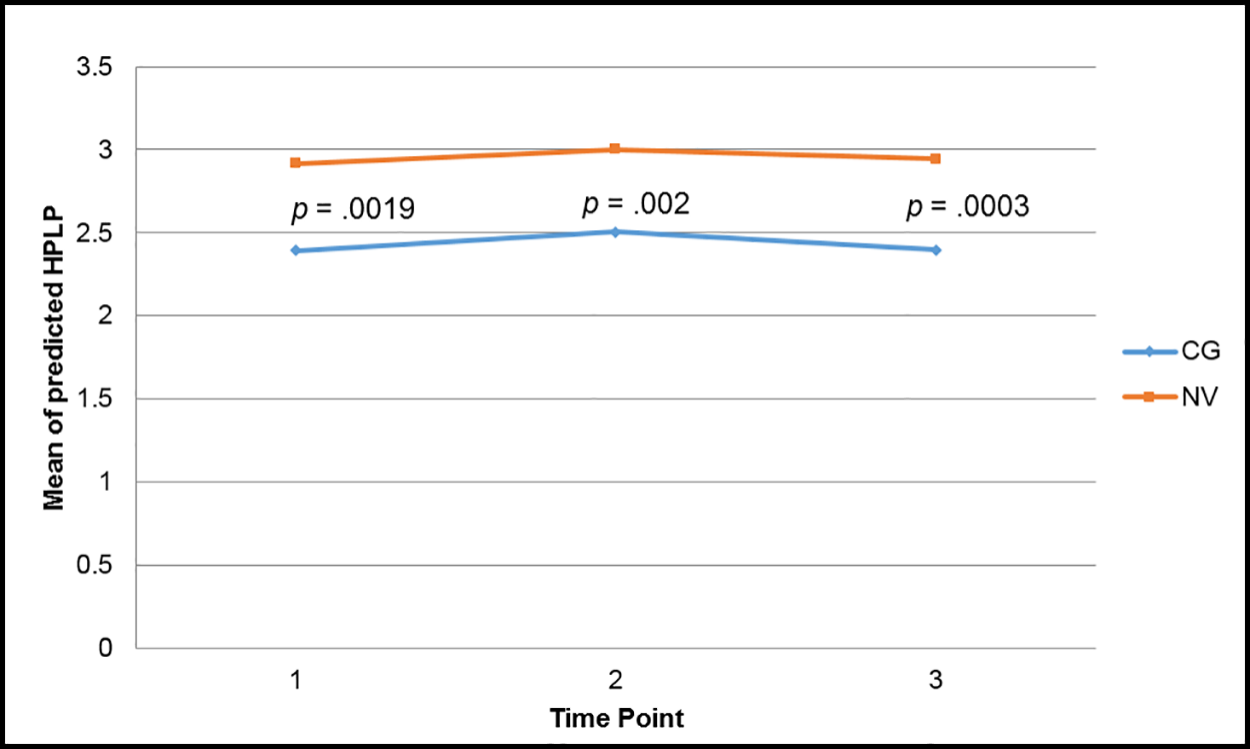
Predicted total scores of HPLP-II over time based on caregiver status. *CG* caregiver, *NV* normal volunteer.

There were no significant baseline differences in health risk behaviors (alcohol consumption and smoking), nor were there any changes in these behaviors over time in either group. Baseline total scores for the HPLP were significantly lower in the caregivers than controls (*p* = .002) (Fig 4). With the exception of nutrition, caregivers scored significantly worse than controls in all of the subscales of the HPLP including physical activity (*p* = .007), interpersonal relationships (*p* = .005), spirituality (*p* = .004), stress reduction (*p* = .004), and health responsibility (*p* = .004). The trajectories of health behaviors did not change significantly over time in either group, and remained significantly different between the two groups over all time points (Fig 4). There were no significant relationships at any time point between total HPLP scores and any of the measures of NMR lipoprotein particle size or concentration.

## Discussion

The findings of this study suggest that traditional measures of cardiometabolic risk may not be robust enough to identify at-risk individuals early in the trajectory of caregiving, while NMR lipoprotein particle size and concentration may be sensitive enough to detect such changes. Three of these more sensitive markers of cardiometabolic risk differed between groups. Levels of HDL-P placed both caregivers and non-caregivers at higher cardiometabolic risk at baseline. While levels of HDL-P improved in the caregivers, these levels remained in the higher cardiometabolic risk category. However, the findings with the VLDL assays were more compelling. Prior to the transplant, caregivers’ levels of large VLDL-P and VLDL-Z were similar to the controls, but these levels worsened over time in the caregivers, while levels in the non-caregivers remained unchanged.

The increasing levels of large VLDL-P and VLDL-Z seen in the HSCT caregivers in this study may be evidence of very early signs of CVD in this population. Unlike the caregivers in this study, none of whom were in the high-risk category for CVD based on their Reynolds Risk Scores, von Kanel et al [9] found that dementia caregivers exhibited FRS that placed them at high risk for CVD. While no direct comparisons can be made in CVD risk between these two groups of caregivers because the scales upon which risk were calculated differ, SBP and DBP as well as total cholesterol were lower and HDL-C was higher in the HSCT caregivers than the dementia caregivers. Perhaps the reason that the dementia caregivers appear to be doing worse in regards to cardiometabolic risk than the HSCT caregivers can be attributed to the fact that the dementia caregivers had been enduring the effects of caregiving for up to twelve years. While HSCT caregiving is stressful and demanding, the caregivers in this study may not yet have experienced the chronic, long-term stress of caregiving that appears to be the nature of dementia caregiving. Furthermore, the mean age of the dementia caregivers was over 72 years, twenty years older, on average, than the caregivers in this study. It is possible that the HSCT caregivers in this study may be in the early stages of the development of CVD and have not yet experienced the same effects of aging and chronic caregiving stress on their cardiovascular health that were seen in dementia caregivers.

Although the immediate period around the transplant procedure is often the most stressful, the impact on the patient and caregiver is far from over when they leave the acute care facility. The majority of HSCT recipients survive beyond two years, but many experience lingering health effects and unique complications such as chronic GVHD that lead to chronic physical and psychosocial problems for the survivor as well as the caregiver [36–38]. Thus, while some caregivers may recover from the stress of the acute HSCT experience with no long-term health consequences, many will continue to experience the stress and uncertainty of caregiving for many years [39].

Despite the increasing levels of large VLDL-P and VLDL-Z along the caregiving trajectory, neither LDL-C nor the NMR lipoprotein measures of LDL-C (LDL-P, LDL-Z) explained CVD risk in the caregivers in this study. The Apolipoprotein B/Apolipoprotein A-1 ratio, an important predictor of coronary death that is associated with metabolic syndrome [40], did not differ in caregivers and controls. LDL-C has received a great deal of attention as a marker of CVD risk and, as a result, has become a major focus of primary prevention of CVD [28]. Lowering LDL-C with statin therapy reduces CVD morbidity and mortality by approximately 20% [41]. Despite the importance of LDL-C as a marker of CVD and a focus of treatment, there remains significant residual risk for CVD that could be accounted for by other lipoproteins. Perhaps the stress of caregiving has a bigger impact on triglyceride-rich remnant lipoproteins such as VLDL, and it is through this pathway that caregivers are at increased cardiometabolic risk. One of the main determinants of circulating triglycerides is the level of lipoprotein lipase, which is well known to be affected by stress [42], as well as by exercise [43]. The fact that measures related to VLDL changed the most in caregivers suggest that, in the future, it may be valuable to also measure post-prandial lipids, particularly given the recent evidence of their importance in cardiovascular disease [43].

Another important finding in this study is that participation in all of the health promoting behaviors except nutrition was worse in the caregivers than controls at all time-points. Compared to control subjects, the HSCT caregivers are not exercising, engaging in stress management, seeking routine medical care, or experiencing the benefits of spirituality and social support. Three quarters of the caregivers were overweight or obese and reported having at least one chronic health condition at the start of caregiving. This scenario may be indicative of a “perfect storm,” as caregivers in this study were coming into the caregiving experience in poor health, experiencing high levels of stress related to caregiving [19], and doing very little to take care of themselves throughout the process.

While this study adds to the evidence regarding cardiometabolic risk in caregivers, it is not without limitations. The small sample size may have contributed to the inability to identify additional differences between the two groups and to see a significant relationship between health behaviors and cardiometabolic risk. While the researchers carefully matched the caregivers and control subjects in age, gender and race/ethnicity, and they controlled for any significant demographic differences such as income and education, the two groups may have differed in other ways that could have influenced study outcomes. While one of only a few research studies to follow caregivers longitudinally, the average length of time that caregivers were followed in was relatively short (approximately 84 days) [19], and this could have contributed to an inability to see differences in traditional measures of cardiometabolic risk. Despite its small sample size and short duration, this study, utilizing more sensitive indicators of cardiometabolic risk, provides evidence of a possible early mechanism in the development of cardiometabolic disease in caregivers that warrants future investigation.

## References

[1] Dimsdale JE (2008) Psychological stress and cardiovascular disease. J Am Coll Cardiol 51: 1237–1246. 10.1016/j.jacc.2007.12.024 18371552 PMC2633295

[2] McEwen BS, Gianaros PJ (2010) Central role of the brain in stress and adaptation: links to socioeconomic status, health, and disease. Ann N Y Acad Sci 1186: 190–222. 10.1111/j.1749-6632.2009.05331.x 20201874 PMC2864527

[3] Hering D, Lachowska K, Schlaich M (2015) Role of the Sympathetic Nervous System in Stress-Mediated Cardiovascular Disease. Curr Hypertens Rep 17: 80 10.1007/s11906-015-0594-5 26318888

[4] Golbidi S, Frisbee JC, Laher I (2015) Chronic stress impacts the cardiovascular system: animal models and clinical outcomes. Am J Physiol Heart Circ Physiol 308: H1476–1498. 10.1152/ajpheart.00859.2014 25888514

[5] Sinha R, Jastreboff AM (2013) Stress as a common risk factor for obesity and addiction. Biol Psychiatry 73: 827–835. 10.1016/j.biopsych.2013.01.032 23541000 PMC3658316

[6] Schulz R, Beach SR (1999) Caregiving as a risk factor for mortality—The caregiver health effects study. Jama-Journal of the American Medical Association 282: 2215–2219. 10.1001/jama.282.23.2215 10605972

[7] Brown SL, Smith DM, Schulz R, Kabeto MU, Ubel PA, Poulin M, et al (2009) Caregiving behavior is associated with decreased mortality risk. Psychol Sci 20: 488–494. 10.1111/j.1467-9280.2009.02323.x 19320860 PMC2865652

[8] Lee S, Colditz GA, Berkman LF, Kawachi I (2003) Caregiving and risk of coronary heart disease in U.S. women: a prospective study. Am J Prev Med 24: 113–119. 12568816 10.1016/s0749-3797(02)00582-2

[9] von Kanel R, Mausbach BT, Patterson TL, Dimsdale JE, Aschbacher K, Mills PJ, et al (2008) Increased Framingham Coronary Heart Disease Risk Score in dementia caregivers relative to non-caregiving controls. Gerontology 54: 131–137. 10.1159/000113649 18204247

[10] Mausbach BT, von Kanel R, Aschbacher K, Roepke SK, Dimsdale JE, Ziegler MG, et al (2007) Spousal caregivers of patients with Alzheimer's disease show longitudinal increases in plasma level of tissue-type plasminogen activator antigen. Psychosom Med 69: 816–822. 10.1097/PSY.0b013e318157d461 17942832

[11] Roepke SK, Allison M, Von Kanel R, Mausbach BT, Chattillion EA, Harmell AL, et al (2012) Relationship between chronic stress and carotid intima-media thickness (IMT) in elderly Alzheimer's disease caregivers. Stress 15: 121–129. 10.3109/10253890.2011.596866 21790484 PMC3223262

[12] Arnaout K, Patel N, Jain M, El-Amm J, Amro F, Tabbara IA (2014) Complications of allogeneic hematopoietic stem cell transplantation. Cancer Invest 32: 349–362. 10.3109/07357907.2014.919301 24902046

[13] Foster LW, McLellan L, Rybicki L, Dabney J, Copelan E, Bolwell B (2013) Validating the positive impact of in-hospital lay care-partner support on patient survival in allogeneic BMT: a prospective study. Bone Marrow Transplant 48: 671–677. 10.1038/bmt.2012.208 23103681

[14] Gibbons SW, Ross A, Bevans M (2014) Liminality as a Conceptual Frame for Understanding the Family Caregiving Rite of Passage: An Integrative Review. Research in Nursing & Health 37: 423–436. 25176315 10.1002/nur.21622 PMC4180249

[15] Bevans M, Sternberg EM (2012) Caregiving burden, stress, and health effects among family caregivers of adult cancer patients. JAMA 307: 398–403. 10.1001/jama.2012.29 22274687 PMC3304539

[16] Bishop MM (2009) Psychosocial sequelae of hematopoietic cell transplantation in survivors and caregivers. Biol Blood Marrow Transplant 15: 29–32. 10.1016/j.bbmt.2008.10.001 19147074

[17] Beattie S, Lebel S (2011) The experience of caregivers of hematological cancer patients undergoing a hematopoietic stem cell transplant: a comprehensive literature review. Psychooncology 20: 1137–1150. 10.1002/pon.1962 21425389

[18] Laudenslager ML (2014) "Anatomy of an Illness": control from a caregiver's perspective. Brain Behav Immun 36: 1–8. 10.1016/j.bbi.2013.08.012 24012646 PMC3947217

[19] Bevans MF, Ross A, Wehrlen L, Klagholz SD, Yang L, Childs R, et al (2016) Documenting stress in caregivers of transplantation patients: initial evidence of HPA dysregulation. Stress 19: 175–184. 10.3109/10253890.2016.1146670 26949170 PMC4976925

[20] Williams LA (2007) Whatever it takes: Informal caregiving dynamics in blood and marrow transplantation 62. Oncology Nursing Forum 34: 379–387. 10.1188/07.ONF.379-387 17573302

[21] Stenberg U, Ruland CM, Miaskowski C (2010) Review of the literature on the effects of caring for a patient with cancer. Psychooncology 19: 1013–1025. 10.1002/pon.1670 20014159

[22] Ross A, Sundaramurthi T, Bevans M (2013) A labor of love: the influence of cancer caregiving on health behaviors. Cancer Nurs 36: 474–483. 10.1097/NCC.0b013e3182747b75 23132126 PMC4196265

[23] Grundy SM (2004) Obesity, metabolic syndrome, and cardiovascular disease. J Clin Endocrinol Metab 89: 2595–2600. 10.1210/jc.2004-0372 15181029

[24] Grundy SM, Brewer HB Jr., Cleeman JI, Smith SC Jr., Lenfant C (2004) Definition of metabolic syndrome: Report of the National Heart, Lung, and Blood Institute/American Heart Association conference on scientific issues related to definition. Circulation 109: 433–438. 10.1161/01.CIR.0000111245.75752.C6 14744958

[25] Rasouli M, Kiasari AM (2006) Interactions of serum hsCRP with apoB, apoB/AI ratio and some components of metabolic syndrome amplify the predictive values for coronary artery disease. Clin Biochem 39: 971–977. 10.1016/j.clinbiochem.2006.07.003 16963013

[26] Ridker PM, Buring JE, Rifai N, Cook NR (2007) Development and validation of improved algorithms for the assessment of global cardiovascular risk in women: the Reynolds Risk Score. JAMA 297: 611–619. 10.1001/jama.297.6.611 17299196

[27] DeFilippis AP, Blaha MJ, Ndumele CE, Budoff MJ, Lloyd-Jones DM, McClelland RL, et al (2011) The association of Framingham and Reynolds risk scores with incidence and progression of coronary artery calcification in MESA (Multi-Ethnic Study of Atherosclerosis). J Am Coll Cardiol 58: 2076–2083. 10.1016/j.jacc.2011.08.022 22051329 PMC4079464

[28] Taylor F, Huffman MD, Macedo AF, Moore TH, Burke M, Davey Smith G, et al (2013) Statins for the primary prevention of cardiovascular disease. Cochrane Database Syst Rev 1: CD004816. 10.1002/14651858.CD004816.pub5 PMC6481400 23440795

[29] Otvos JD, Mora S, Shalaurova I, Greenland P, Mackey RH, Goff DC Jr. (2011) Clinical implications of discordance between low-density lipoprotein cholesterol and particle number. J Clin Lipidol 5: 105–113. 10.1016/j.jacl.2011.02.001 21392724 PMC3070150

[30] Charlson ME, Pompei P, Ales KL, MacKenzie CR (1987) A new method of classifying prognostic comorbidity in longitudinal studies: development and validation. J Chronic Dis 40: 373–383. 3558716 10.1016/0021-9681(87)90171-8

[31] Frazier-Wood AC, Garvey WT, Dall T, Honigberg R, Pourfarzib R (2012) Opportunities for using lipoprotein subclass profile by nuclear magnetic resonance spectroscopy in assessing insulin resistance and diabetes prediction. Metab Syndr Relat Disord 10: 244–251. 10.1089/met.2011.0148 22533466 PMC3409454

[32] Mackey RH, Mora S, Bertoni AG, Wassel CL, Carnethon MR, Sibley CT, et al (2015) Lipoprotein particles and incident type 2 diabetes in the multi-ethnic study of atherosclerosis. Diabetes Care 38: 628–636. 10.2337/dc14-0645 25592196 PMC4370328

[33] Walker SN, Sechrist KR, Pender NJ (1987) The Health-Promoting Lifestyle Profile: development and psychometric characteristics. Nurs Res 36: 76–81. 3644262

[34] Mora S, Otvos JD, Rosenson RS, Pradhan A, Buring JE, Ridker PM (2010) Lipoprotein particle size and concentration by nuclear magnetic resonance and incident type 2 diabetes in women. Diabetes 59: 1153–1160. 10.2337/db09-1114 20185808 PMC2857895

[35] Phillips CM, Perry IJ (2015) Lipoprotein particle subclass profiles among metabolically healthy and unhealthy obese and non-obese adults: does size matter? Atherosclerosis 242: 399–406. 10.1016/j.atherosclerosis.2015.07.040 26277632

[36] Hashmi S, Carpenter P, Khera N, Tichelli A, Savani BN (2015) Lost in transition: the essential need for long-term follow-up clinic for blood and marrow transplantation survivors. Biol Blood Marrow Transplant 21: 225–232. 10.1016/j.bbmt.2014.06.035 24999225

[37] Pavletic SZ, Fowler DH (2012) Are we making progress in GVHD prophylaxis and treatment? Hematology Am Soc Hematol Educ Program 2012: 251–264. 23233589 10.1182/asheducation-2012.1.251

[38] Wulff-Burchfield EM, Jagasia M, Savani BN (2013) Long-term follow-up of informal caregivers after allo-SCT: a systematic review. Bone Marrow Transplant 48: 469–473. 10.1038/bmt.2012.123 22732697

[39] Bishop MM, Curbow BA, Springer SH, Lee JA, Wingard JR (2011) Comparison of lasting life changes after cancer and BMT: perspectives of long-term survivors and spouses. Psychooncology 20: 926–934. 10.1002/pon.1812 20690114

[40] Wallenfeldt K, Bokemark L, Wikstrand J, Hulthe J, Fagerberg B (2004) Apolipoprotein B/apolipoprotein A-I in relation to the metabolic syndrome and change in carotid artery intima-media thickness during 3 years in middle-aged men. Stroke 35: 2248–2252. 10.1161/01.STR.0000140629.65145.3c 15345795

[41] Baigent C, Keech A, Kearney PM, Blackwell L, Buck G, Pollicino C, et al (2005) Efficacy and safety of cholesterol-lowering treatment: prospective meta-analysis of data from 90,056 participants in 14 randomised trials of statins. Lancet 366: 1267–1278. 10.1016/S0140-6736(05)67394-1 16214597

[42] Peckett AJ, Wright DC, Riddell MC (2011) The effects of glucocorticoids on adipose tissue lipid metabolism. Metabolism 60: 1500–1510. 10.1016/j.metabol.2011.06.012 21864867

[43] Plaisance EP, Grandjean PW, Mahurin AJ (2009) Independent and combined effects of aerobic exercise and pharmacological strategies on serum triglyceride concentrations: a qualitative review. Phys Sportsmed 37: 11**–**19. 10.3810/psm.2009.04.1678 20048483

